# The use of PET/MRI in radiotherapy

**DOI:** 10.1186/s13244-024-01627-6

**Published:** 2024-02-27

**Authors:** Qi Yan, Xia Yan, Xin Yang, Sijin Li, Jianbo Song

**Affiliations:** 1 Cancer Center, Third Hospital of Shanxi Medical University, Shanxi Bethune Hospital, Shanxi Academy of Medical Sciences Tongji Shanxi Hospital, Taiyuan, China; 2 Cancer Center, Shanxi Bethune Hospital, Shanxi Academy of Medical Sciences, Tongji Shanxi Hospital, Third Hospital of Shanxi Medical University, Taiyuan, China; 3 Shanxi Provincial Key Laboratory for Translational Nuclear Medicine and Precision Protection, Taiyuan, China; 4 Department of Nuclear Medicine, First Hospital of Shanxi Medical University, Shanxi Medical University, Taiyuan, China

**Keywords:** PET/MRI, Radiotherapy, Oncology, Integrated imaging, Molecular imaging

## Abstract

Positron emission tomography/magnetic resonance imaging (PET/MRI) is a hybrid imaging technique that quantitatively combines the metabolic and functional data from positron emission tomography (PET) with anatomical and physiological information from MRI. As PET/MRI technology has advanced, its applications in cancer care have expanded. Recent studies have demonstrated that PET/MRI provides unique advantages in the field of radiotherapy and has become invaluable in guiding precision radiotherapy techniques. This review discusses the rationale and clinical evidence supporting the use of PET/MRI for radiation positioning, target delineation, efficacy evaluation, and patient surveillance.

**Critical relevance statement** This article critically assesses the transformative role of PET/MRI in advancing precision radiotherapy, providing essential insights into improved radiation positioning, target delineation, efficacy evaluation, and patient surveillance in clinical radiology practice.

**Key points**

• The emergence of PET/MRI will be a key bridge for precise radiotherapy.

• PET/MRI has unique advantages in the whole process of radiotherapy.

• New tracers and nanoparticle probes will broaden the use of PET/MRI in radiation.

• PET/MRI will be utilized more frequently for radiotherapy.

**Graphical Abstract:**

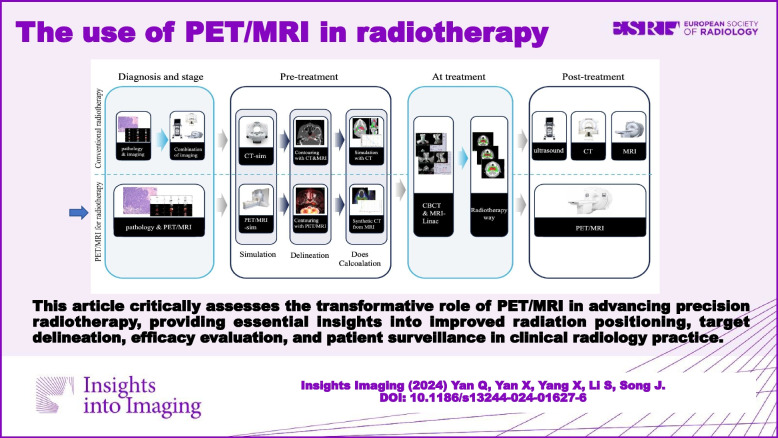

## Introduction

**Fig. 1:**
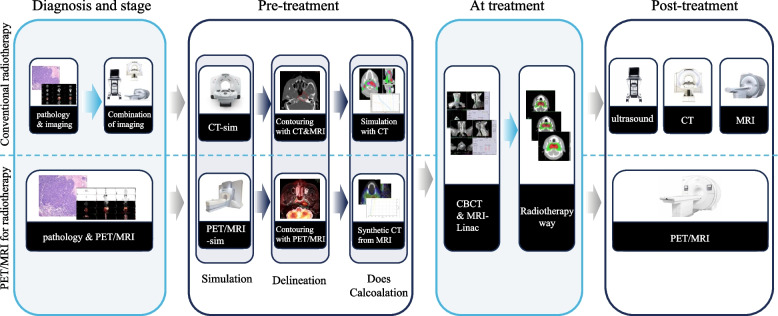
Comparison of PET/MRI for radiotherapy procedures with conventional radiotherapy procedures

Radiotherapy is a standard component of care for many cancer patients. With the advancement of radiotherapy technology, three-dimensional conformal radiotherapy (3D-CRT), intensity modulated radiation therapy (IMRT), stereotactic radiotherapy (SRT), and tomotherapy have arisen and dramatically enhanced the prognosis of cancer patients [1, 2]. CT is widely used in radiation planning due to its ability to provide high-resolution anatomical information, while MRI is valued for its excellent soft tissue contrast [3, 4]. Nevertheless, standard imaging techniques only reveal morphological alterations, which is insufficient for precise radiation planning. To overcome this limitation, researchers have been exploring the use of multimodal imaging to enhance its precision. One promising avenue is the integration of multi-parametric PET/MRI into one-stop-shop radiotherapy (RT) planning workflow (Fig. 1).

PET/MRI is a hybrid imaging technique that quantitatively combines metabolic functional data from PET with anatomical and physiological information from MRI [5, 6]. Due to its high soft tissue contrast, PET/MRI may be extremely accurate in T-staging of cancer, such as head and neck, abdominal, and pelvic tumors. Recent studies have begun to show that it is also valuable in treating lymphoma, liver, and breast cancer [7–9]. When it comes to finding lymph node metastases and faraway metastases, PET/MRI is also superior to CT, MRI, and PET/CT, as PET/MRI can provide dynamic enhanced imaging, diffusion-weighted imaging (DWI), and signal strength (SI) assessment that can display non-fluorodeoxyglucose (non-FDG) uptake lesions and avoid misdiagnosis of sites with physiologic FDG uptake, such as the adrenal glands and mucous membranes [10–13]. Thus, given the consideration of the advantages mentioned, the efficacy of PET/MRI in tumor diagnosis and staging has been the subject of extensive research [14–16]. Recently, increasing researches indicate that PET/MRI can also be utilized in radiotherapy and can further improve the accuracy of radiation therapy [17–21]. This review summarizes the available literature on the utilization of PET/MRI for radiation placement, target delineation, efficacy evaluation, and patient surveillance (Table 1).

**Table 1:** Comparison of PET/MRI and other conventional image-base modality in radiotherapy

	**CT**	**MRI**	**PET/CT**	**PET/MRI**
**Radiotherapy planning**	Fused with other images for target delineation Providing tissue electron density information directly for dose calculation	Fused with other images for target delineation Co-registration with CT images or synthesis of pseudo-CT images for dose calculation	BTV provided by PET assist in GTV delineation Providing tissue electron density information directly for dose calculation	BTV provided by PET assist in GTV delineation Co-registration with CT images or synthesis of pseudo-CT images for dose calculation
**Treatment evaluation**	Assessing the treatment effect from tumor morphology Not available for pregnant women or children	Assessing the treatment effect from tumor morphology and parameter changes Not available for patients with metal implants, ferromagnetic objects	Assessing the treatment effect from anatomical and functional information Not available for pregnant women or children	Assessing the treatment effect from anatomical, functional information and parameter changes Not available for patients with metal implants, ferromagnetic objects
**Patient surveillance**	Having ionizing radiation Acquisition quickly	No-ionizing radiation Long acquisition	Having ionizing radiation Long acquisition	No-ionizing radiation Long acquisition

## Radiotherapy positioning

Accurate radiotherapy positioning is a critical component of the pre-radiotherapy preparation process. The advent of ^18^F-fluorodeoxyglucose ([^18^F]-FDG) PET/MRI has provided clinicians with more precise and comprehensive imaging data, enhancing the effectiveness of patient positioning for radiotherapy. However, alongside this technology, specialized positioning devices have been developed and introduced which, while improving precision, also present new challenges in terms of integration and optimization.

### Specific equipment and material

In addition to the standard diagnostic [^18^F]-FDG PET/MRI, the [^18^F]-FDG PET/MRI used in radiotherapy incorporates specialized equipment and a method for external-internal reference point positioning (Table 2). The radiation equipment consists of a flat table and a patient positioning device. As for the treatment bed, unlike the curved one used in diagnostic MR, it is flat in [^18^F]-FDG PET/MRI for radiotherapy. This is because, on the one hand, [^18^F]-FDG PET/MRI images need to be aligned with CT images for accurate dose calculation. In CT, the bed used for treatment is flat. If a curved treatment bed is still used, the image quality will be affected. On the other hand, patients need to be placed in the same way every time they get radiotherapy through fixed devices, which are often installed on a flat structure [22]. In reference point positioning systems, isocentric placement is mostly done with the help of laser systems [21], where markers are made on patients’ skin to ensure accurate repositioning. However, when the positioning fixture is included, the anterior receiver coil of the diagnostic machine is not compatible with the radiation equipment. Thus, a radiotherapy-specific coil and coil holder were also introduced. In this way, the near-coil effect, that the signal increases as the distance from the coil decreases, was minimized [18]. However, due to technical restrictions, the whole-body coil holder may cause MR image distortion and truncation. To restore truncations, PET-based and purely MR-based methods have been introduced [21, 23, 24].

**Table 2:** Comparison of diagnostic PET/MRI and PET/MRI simulator for radiotherapy positioning

	**Diagnostic PET/MRI**	**PET/MRI simulator**
**Purpose**	Exploration, qualitative evaluation, staging, assisted target delineation, efficacy evaluation and prognosis prediction	To provide imaging data for target volume delineation in the 3D direction
**Positioning device**	Does not involved	Positioning devices such as thermoplastic masks and laser system are used to fix the patient’s position
**Anterior receiver coil**	RF coil could touch and deform the surface of the patients	RF coil was fixed by the coil holder away from the patient’s surface, ensuring a gap for the placement of the positioning device and repeatability of each positioning for the patient
**Treatment bed**	Diagnostic PET/MRI is the curved one	The PET/MRI simulator is the flat one
**Image quality**	MRI image: • No positioning device • Almost no effect with coil • A negligible effect with treatment bed PET image: • No positioning device • A slight effect with coil • A slight effect with treatment bed	MRI image: • A slight effect with positioning device • No image distortion with coil and coil holder • A negligible effect with treatment bed PET image: • Almost no effect with positioning device • A slight effect with coil and coil holder • A slight effect with treatment bed

**Fig. 2:**
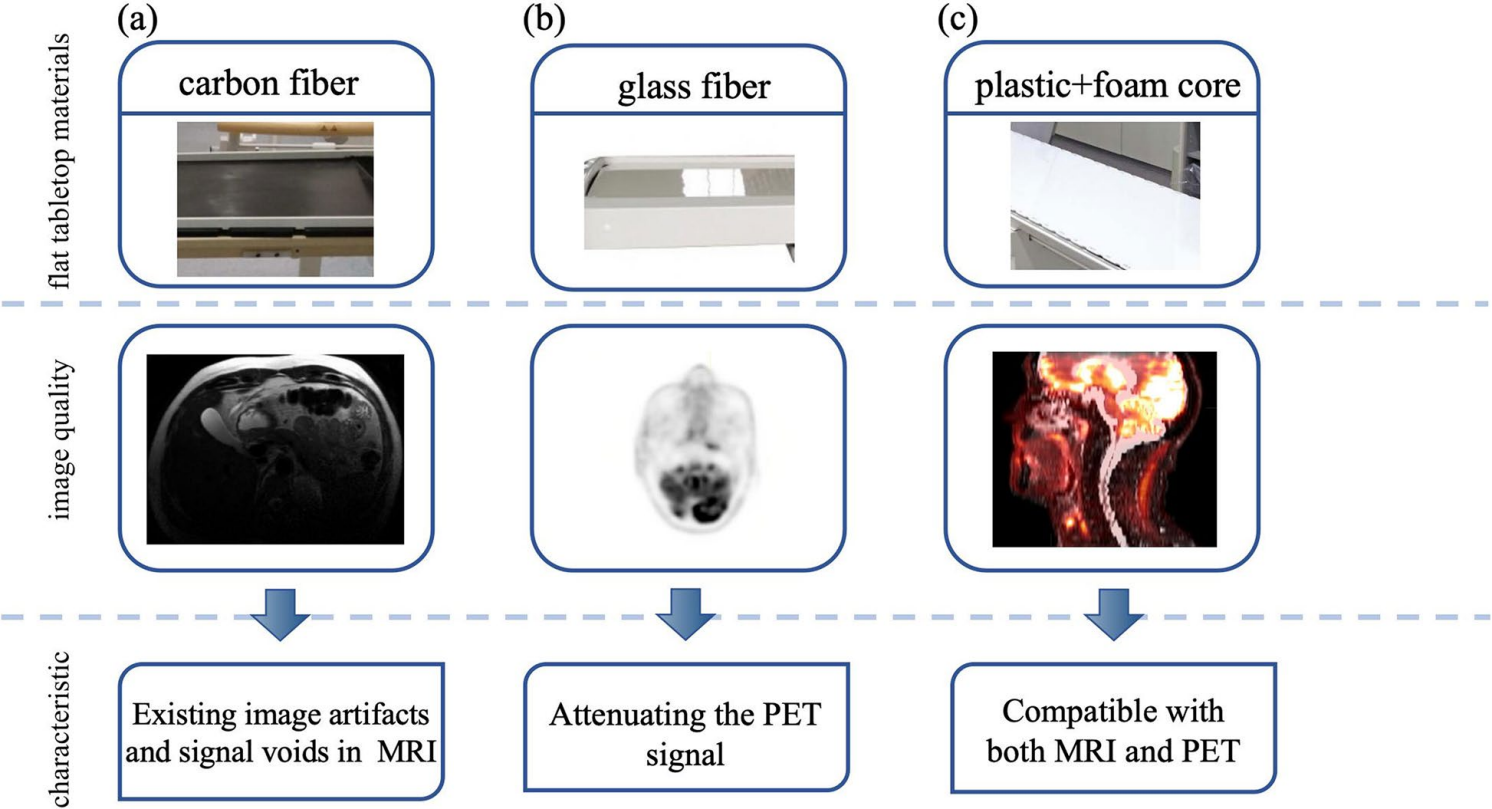
The difference between image quality obtained from different types of tabletop materials using (**a**) carbon fiber, (**b**) glass fiber, and (**c**) plastic sandwich with foam core

Materials in radiotherapy equipment are also critical as they directly impact the quality of imaging. Existing materials for flat tabletops are primarily composed of carbon fiber which offers minimal photon attenuation. However, the conductive nature of carbon fiber can generate surface eddy currents, leading to the production of image artifacts and signal voids, making it incompatible with MRI. Subsequently, flat tabletops made of glass fibers were introduced; however, glass fibers significantly attenuate the PET signal, leading to artifacts and increased quantization errors in PET images, rendering them incompatible with PET [25–27]. Based on this, materials combining a plastic sandwich with a foam core have been developed, which are designed to have reduced and homogeneous photon attenuation, making them compatible with both MRI and PET [21] (details shown in Fig. 2). For the MR coil, because the gamma-ray attenuation of materials is closely tied to the electron density distribution of those materials, the use of electron-dense materials is not an option [28]. Building on this principle, the use of thin plastic shells, thin copper wire coils, and lightweight coil technologies are being explored to reduce PET attenuation and enhance image quality [29, 30].

### Effect on image quality

With the introduction of specific equipment and material for [^18^F]-FDG PET/MRI, the image quality of [^18^F]-FDG PET/MRI may be affected. The signal-to-noise ratio (SNR) is a critical metric for evaluating the image quality of MRI. Witoszynskyj et al. reported mean SNR values at the slice through the center of the phantom were 98.0 ± 27.1 with a flat table and 98.3 ± 27.0 without it, suggesting that the flat table has a negligible effect on SNR [31]. In addition, as positioning devices increase the distance between the patient and the coil, the total SNR of MRI pictures was reduced by 25% compared with the usual setup [21]. Winter et al. observed a slight reduction in image SNR with the radiotherapy device compared to diagnostic [^18^F]-FDG PET/MRI. However, the target volume remained consistent with that delineated by MRI, indicating that the reduction in SNR may not pose a limitation in RT planning [17]. However, improved image quality is still required for further precision radiotherapy. Although the SNR can be improved in several ways, the process of doing so often comes with other problems. For instance, by changing the MR parameters, such as raising the signal average [32], lowering the acceleration, or shortening the time of echo (TE), the SNR can be increased, but the acquisition time will also be increased [17]. Additionally, an adjustable coil bridge was developed to solve the problem of patient-coil distance, and while moving the coil closer to the patient improves image quality, positioning repeatability may become more difficult [33]. Boosting spatial resolution, which defines the picture’s capacity to portray fine structures, is another technique for improving MRI image quality, but care must be given not to impair SNR due to the inverse relationship between spatial resolution and SNR [17]. Recently, a new noise reduction and reconstruction technique using deep learning reconstruction, which only changes the SNR, has emerged, but its accuracy needs to be further verified [34].

In PET imaging, objects within the field of view (FOV) of the PET detector can attenuate and scatter PET photons, leading to reduced image quality. Thus, thin patient positioning devices, such as thermoplastic masks, were reported and found to have a negligible effect on PET quantification [21]. Witoszynskyj et al. demonstrated, using a phantom, that image activity near the flat tabletop side was underestimated, but this can be rectified by employing attenuation correction [31]. Based on phantom and human data, Paulus et al. predicted that accounting for the coil holder, including the radiofrequency (RF) coil, in the attenuation correction would reduce the deviation from 13.8 to 0.8% [21].

### Attenuation correction (AC)

[^18^F]-FDG PET/MRI positioning images, unlike diagnostic images that only need to provide qualitative information, will subsequently be used for dose calculations in radiation therapy and thus require an accurate AC map. The introduction of coil fixation devices in radiation equipment mitigates the problem of AC map error caused by the unfixed position and geometric shape of the flexible coil during scanning [32, 35, 36]. But another key issue arises in [^18^F]-FDG PET/MRI, different from PET/CT, MRI cannot provide photon attenuation data that can be directly used for AC of PET. To resolve this issue, different proposals have been suggested, including MR-based image segmentation, atlas-based AC, and substitute CT (s-CT) [37–40]. MR-based image segmentation divides the attenuated images into four categories, but bone segmentation is not performed due to the low intensity and non-specific signals of cortical bone on MRI [37]. Atlas-based AC can take into account cortical bone. However, the registration of the whole-body image to the atlas is prone to errors due to the different stiffness of the chest and abdomen and the head [41, 42]. s-CT correlates the voxel values of CT with MRI images, which can turn the MRI image into s-CT, and automatically generates attenuation maps with more fine intervals than those generated by segmentation [40]. Recently, artificial intelligence (AI) employing deep learning convolutional neural networks (CNNs) is emerging as an alternative to traditional AC methods owing to its speed, accuracy, and robustness [43]. In addition, different AC methods were generated according to the characteristics of different components. Rigid hardware components, like coil holders and flat tabletops, can undergo AC using CT-based 3D attenuation maps or ^68^Ge/^68^Ga projection scanning attenuation maps, due to their fixed positions and strict geometry [44]. In contrast, AC for flexible coils can be achieved using MRI with ultra-short echo time (MRI-UTE) sequences. However, this method has very limited visualization and provides insufficient information on the coil components for accurate AC [44, 45]. The marker-based correction method, automatically determining the position of surface coils using markers, was proposed for accurate AC [46]. Moreover, Lindemann et al. proposed a method based on computer-aided design (CAD), which generated AC-map without artifacts and improved spatial resolution [31, 32, 46, 47].

Once the attenuation map is obtained, it is vital to assess its repeatability during the radiation repositioning process. Since the [^18^F]-FDG PET/MRI flat tabletop remains in a fixed position, only a single registration is necessary. However, as the coil holder undergoes repeated installation and removal, it is essential to confirm the accuracy of the attenuation map during repositioning [21]. Paulus et al. evaluated the accuracy of the coil holder during multiple repositionings using a phantom and an active ^68^Ge rod source. They found that there were only slight deviations in accuracy when the RF coil holder was repeatedly moved without altering the phantom’s position, suggesting that the attenuation map of the coil holder is reliable for repositioning purposes [21].

### MRI protocols for RT positioning

In the context of RT localization using MRI, the selection of sequences is a crucial step in ensuring both efficiency and accuracy. Typically, T1-weighted imaging (T1WI), T2-weighted imaging (T2WI), and DWI sequences are chosen. T1WI and T2WI offer essential anatomical information for registration and target delineation. DWI, providing valuable metabolic insights, aids in refining tumor boundaries and assessing lymph node involvement [48]. Recently, in order to further optimize the scanning time of MRI sequences, and to improve patient compliance while ensuring accurate clinical information, different sequences are recommended for different lesions [48–54]. For instance, inversion recovery gradient echo (IR-GRE) is commonly used for T1WI in brain tumors, while turbo spin-echo (TSE) and fluid-attenuated inversion recovery (FLAIR) imaging are frequently employed for T2WI [51, 52]. In areas with abundant soft tissues like the head and neck, breast, abdomen, and pelvis, fat saturation technology is often applied for improved tumor and organ visualization [48, 53, 54].

## Radiotherapy planning

With the introduction of the IMRT, the significance of accurate tumor volumes is re-emphasized. Since [^18^F]-FDG PET/MRI can concurrently capture both tissue morphology and tumor metabolism information, it can accurately distinguish tumor tissue from neighboring tissues, making it valuable for accurate target identification in radiotherapy [55].

### Target delineation

Gross tumor volume (GTV) is commonly outlined using the tumor morphology visualized on MRI. However, integrating tumor biology information from PET can mitigate the risk of marginal loss, potentially impacting GTV changes. Zhang et al. observed an increasing difference between GTV-MRI and GTV-PET with growing tumor volumes [56, 57]. Notably, while around 90% of GTV-PET overlapped with GTV-MRI, 10% of the tumor and lymph node volume was exclusively identified by PET, emphasizing the value of a combined [^18^F]-FDG PET/MRI approach in RT planning [58]. The emergence of PET/MRI has great hope to achieve accurate target delineation.

**Fig. 3:**
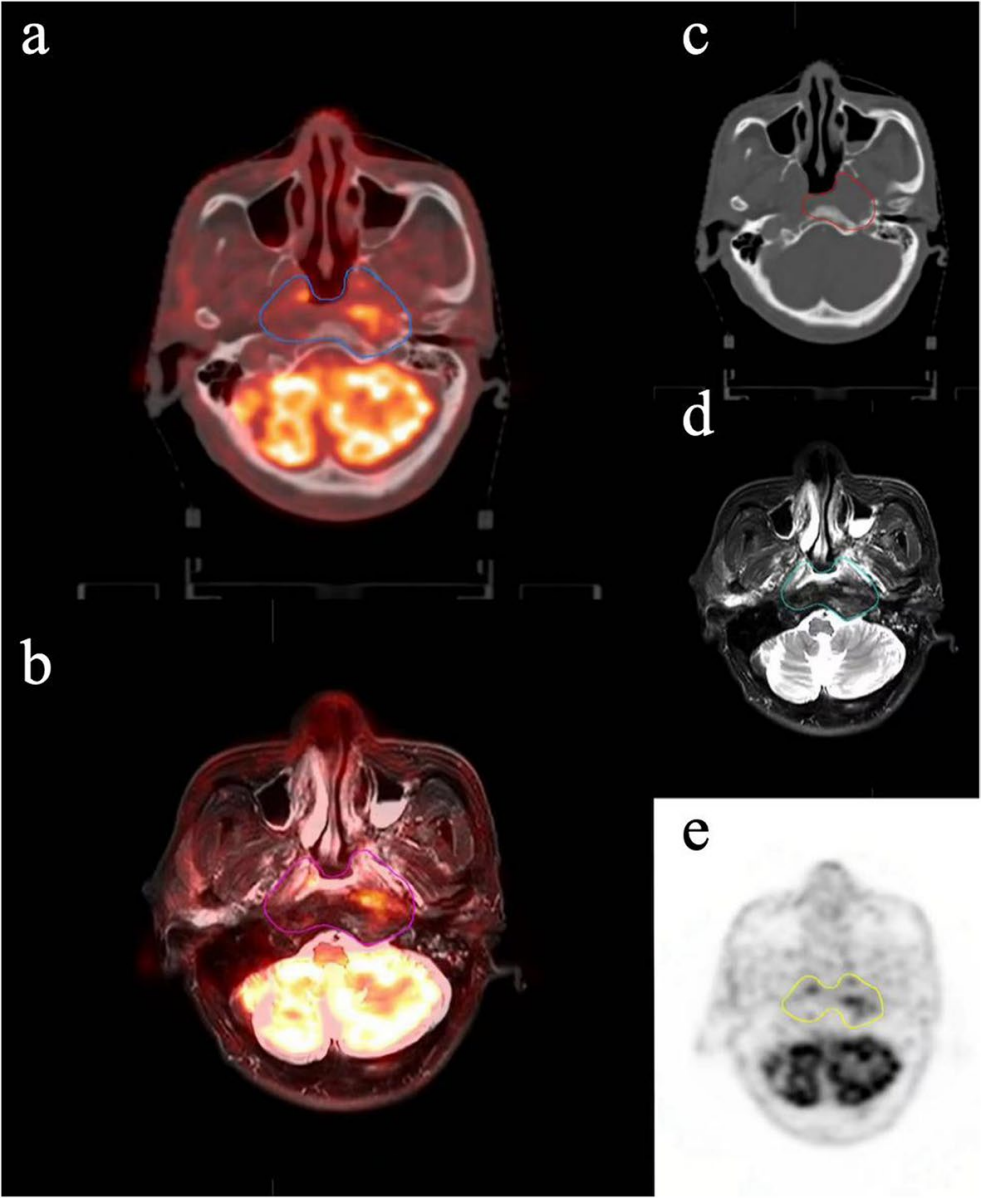
Sixty nine-year-old female with nasopharynx cancers. **a** The blue line represents gross tumor volume (GTV)-PET/CT. **b** The pink line represents GTV-PET/MRI. **c** The red line represents GTV-CT. **d** The green line represents GTV-MRI. **e** The yellow line represents GTV-PET

The effect of PET/MRI on GTV has been studied through the observation of the differences among GTV-MRI, GTV-PET, and GTV-PET/MRI [55, 58, 59]. Using GTV-MRI as a reference, Zhang et al. evaluated the differences between GTV-PET and GTV-[^18^F]-FDG PET/MRI in the delineation of colorectal liver metastases and found that GTV-[^18^F]-FDG PET/MRI had the highest tumor volume [55]. In contrast, Mahase’s work came to the opposite [59], where they found that the average volume of GTV-[^68^Ga]-DOTATATE PET/MRI was lower than that of GTV-MRI in patients with intracranial meningiomas. This discrepancy may be attributed to the utilization of specific tracers in PET, which enable the detection of microscopic tumor activity even in the absence of morphological changes. As a result, the obtained GTV will expand [59, 60]. Conversely, [^68^Ga]-DOTATOC PET/MRI allows for the visualization of the boundary between the tumor and normal tissue, as well as the necrotic parts of the tumor resulting from adjuvant treatment, which can lead to a reduced estimation of GTV [61]. Furthermore, Mahase et al. observed that the use of [^68^Ga]-DOTATATE PET/MRI, which can result in a reduction of GTV, consequently leading to a decrease in both clinical tumor volume (CTV)—comprising the GTV and potentially invaded tissues and planning target volume (PTV), which is derived from the expansion of CTV [59]. In addition, in the lymph node metastasis delineation, the nodal GTV (GTVnd) delineated based on PSMA PET/MRI contains metastatic pelvic lymph nodes more accurately than MRI [62]. However, Liu et al. showed that the CTVs of PSMA PET/MRI and MRI are similar in the target delineation of pelvic lymph node metastasis [62], where the distinction brought about by GTVnd is not apparent, possibly because CTV encompasses a larger range than GTVnd. Moreover, the pathological analysis also confirmed the advantage of PET/MRI in the target delineation, Zhang et al. proposed that PET/MRI is more adept at mapping GTV by comparing the Dice Similarity Coefficient (DSC) of PET/MRI and PET. Additionally, they demonstrated that the longest tumor length measured by PET/MRI correlated well with the longest tumor length measured by histopathological analysis, providing robust evidence for the superior accuracy of GTV delineation by PET/MRI [60, 62]. Figure 3 illustrates the difference between [^18^F]-FDG PET/MRI and other traditional imaging methods for GTV delineation. However, although the advantages of PET/MRI in target delineation have been identified, the impact of PET/MRI on treatment response and survival has not been determined, and further exploration is needed in the future.

In addition to the abovementioned effects of PET/MRI on the target area, manual delineation of tumor volume introduces considerable potential for inter-observer variability [63], which can be mitigated through the application of standardized segmentation techniques. Co-segmentation based on [^18^F]-FDG PET/MRI has garnered significant interest in recent years [64–66]. Leibfarth et al. found that the tumor volume delineated manually by the observer was comparable to that automatically delineated by the [^18^F]-FDG PET/MRI method, indicating the viability of [^18^F]-FDG PET/MRI co-segmentation [66]. In addition, [^18^F]-FDG PET/MRI can provide more stable imaging [55, 67]. Cavaliere et al. demonstrated a high degree of agreement between two groups of observers in the anatomical localization and extent of lesions when using [^18^F]-FDG PET/MRI [68]. Moreover, the DSC of the target volume delineated by different observers on [^18^F]-FDG PET/MRI is greater than that on MRI, demonstrating a higher degree of overlap and less degree of discrimination. To address observer inconsistency and enhance accuracy, some studies propose the use of an adaptive threshold level (aTL) derived from individual maximum standardized uptake values (SUV_max_) [58, 69–71]. It is important to note that the appropriate threshold is not a fixed value due to the low spatial resolution of PET and the existing partial volume effect. Published articles indicate that in lung cancer, thresholds based on a percentage of SUV_max_ range from 15 to 50% [72]. Alfano et al. revealed that the sensitivity and specificity could be improved using a 67% SUV_max_ threshold and 81% SUV_max_, respectively [73]. In addition, Dietlein et al. observed that thresholds may vary depending on the chosen radiotracer [74]. Research is needed to further explore whether the threshold is related to additional tumor-related factors such as hypoxia, proliferation, and histological differences. Despite ongoing innovations [65, 66, 75], errors in automatically delineated target areas may still occur due to imprecise tumor boundaries, necessitating manual adjustments.

### RT dose optimization

The integration of PET in radiotherapy has led to the development of the concept of biological target volume (BTV), which is based on the biological characteristics of tumors. As BTV can furnish insights into the radiation sensitivity of tumors, it is instrumental in determining the appropriate RT dosage [76]. Utilizing the data derived from BTV, higher RT doses are administered to treatment-resistant regions of the tumor, while lower doses are allocated for treatment-sensitive regions [25, 77]. This heterogeneous RT dose distribution strategy not only ensures that a high local dose is delivered to the tumor but also safeguards vital organs, such as the spinal cord and lungs, consequently mitigating the toxic effects on normal tissues [78].

### RT dose calculation

**Fig. 4:**
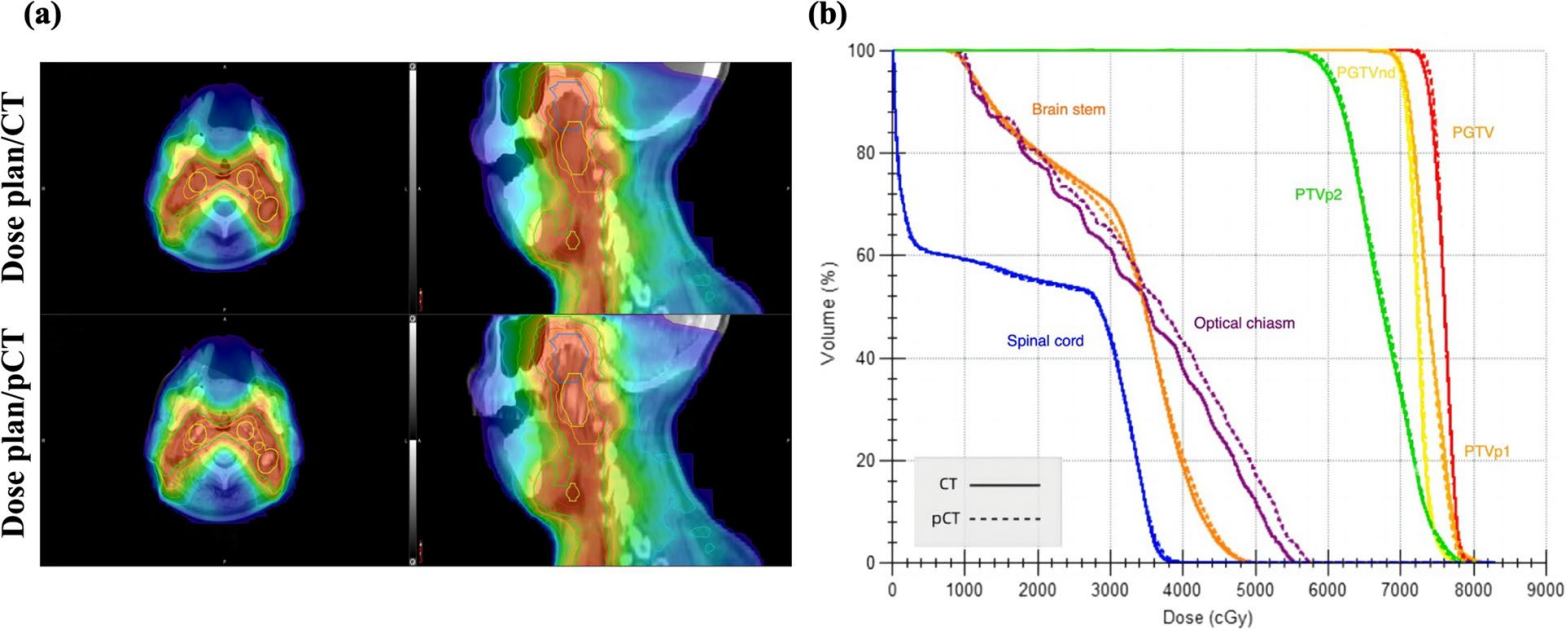
**a** The calculated dose plan for CT and pseudo-CT (pCT) images. **b** Dose and volume histogram (DVH) curve comparing the planned dose of target volumes and different organ at risk (OAR) for CT and pCT

Once the target volume is identified, a dose prescription map becomes essential for dose calculation. Unlike CT, [^18^F]-FDG PET/MRI lacks the capability to acquire tissue electron density values, dose prescription maps of [^18^F]-FDG PET/MRI are typically derived through the co-registration of [^18^F]-FDG PET/MRI and CT images [36, 79]. To streamline the workflow and minimize additional radiation exposure from CT scans, recent advancements suggest the use of pseudo-CT methods generated directly from MR images for dose calculation (Fig. 4) [19, 80, 81]. Ahangari et al. pioneered the transfer of the delineated PTV and optimized treatment plan from CT to pseudo-CT. The dosimetric analysis of the pseudo-CT revealed a mean absolute error within the PTV of 0.17 ± 0.12 Gy, demonstrating a negligible dose difference between the two. This finding was further corroborated by Farjam et al. [19, 82]. In addition, emerging studies indicate that deep learning techniques enable the formulation of pseudo-CT for dose calculation in [^18^F]-FDG PET/MRI, facilitating the swift acquisition of dose prescription while preserving image quality, though the data is still limited [19, 80, 81, 83, 84].

## Treatment evaluation

In clinical practice, CT and MRI are commonly employed to gauge changes in tumor size post-therapy to evaluate the treatment response. However, as morphological alterations in tumors tend to lag behind hypometabolic changes [85], relying on anatomical data for predicting and assessing treatment response may be inaccuracies. Using specific PET tracers and MRI sequences, [^18^F]-FDG PET/MRI is capable to capture both tissue morphology and tumor metabolism, making it significant in treatment outcome prediction and evaluation.

## Pre-treatment evaluation

The prognosis of advanced cancer patients after treatment is still poor, so it is important to accurately evaluate the treatment effect before treatment, and to guide clinicians in choosing the most appropriate treatment strategies. Metabolic tumor volume **(**MTV) and total lesion glycolysis (TLG) are robust [^18^F]-FDG PET/MRI parameters that effectively represent the metabolic activity of tumor cells, providing a comprehensive measure of the overall initial tumor burden before treatment [86]. Meanwhile, as DWI captures the texture of biological tissues [87], the study by Freihat et al. revealed that elevated ADC values derived from DWI, TLG, and MTV before treatment correlated with increased likelihood of recurrence and decreased response rate. This underscores the significance of imaging parameters as valuable predictive biomarkers before treatment, offering more precise information for treatment selection [88]. Recently, image analysis methods such as radiomics and AI have demonstrated their effectiveness in predicting therapy response and prognosis. Features derived from [^18^F]-FDG PET/MRI radiomics have proven to be viable non-invasive imaging biomarkers for predicting efficacy in cervical cancer treatment. However, additional clinical data are required to validate its feasibility in future applications [89, 90].

## Mid-treatment evaluation

To avoid accelerated tumor growth, increased organ toxicity, and increased mortality due to delayed adjustment of therapy [91, 92], it is necessary to evaluate the therapeutic effect and risk of recurrence in the middle of treatment [93, 94]. SUV_max_ and diffusion-related coefficient, which show glucose metabolism and water diffusion during treatment, can assess tumor sensitivity to the treatment by changes in these parameters. Xu et al. discovered that the minimum diffusion correlation coefficient (*D*_min_) and SUV_max_ would be significantly different mid-treatment [86]. In addition, the percentage changes of the maximum standardized uptake value (ΔSUV_max_) and the mean diffusion-related coefficient (ΔD_mean_) of the non-complete metabolic responder during treatment also had a substantial predictive potentiality. Further analysis indicated that the percentage changes in minimum diffusion-related coefficient (ΔD_min_) was corrected with ΔSUV_max_, suggesting that the two related parameters of [^18^F]-FDG PET/MRI could be used as substitutes for each other [86]. However, although investigations have shown that [^18^F]-FDG PET/MRI is useful in treatment evaluation, there is no consensus on the optimal scanning time point [86, 95, 96]. Mayerhoefer et al. demonstrated that capturing treatment-induced changes in lymphoma patients at 48–72 h after the start of treatment could be used to evaluate the therapeutic effect of the first week of treatment [95], while Vojtíšek et al. recorded the pre- and the mid-treatment parameters at week 5 during treatment to predict failure to achieve complete metabolic remission (CMR).

## Post-treatment evaluation

The combination of multiple parameters obtained by different image methods is found to be significantly better for evaluating the therapeutic effect [86, 93, 97, 98]. The advent of PET/MRI enables the simultaneous acquisition of multiple parameters while mitigating errors caused by patients’ movement between different scanning modes, thus reducing the impact of physiological changes in tumor tissue due to time intervals [99]. Romeo et al. conducted a study involving patients with head and neck cancer undergoing concurrent chemoradiotherapy. They found that DCE parameters, such as volume transfer constant (*K*^trans^), and rate constant (*K*_ep_), can confirm the treatment response according to morphological, metabolic, and diffusion data. In addition, in combination with elevated post-treatment SUV_max_, it can help identify patients with disease progression at follow-up who were initially classified as either in partial response (PR) or stable disease (SD) [100–102]. Recently, [^18^F]-FDG PET/MRI has also been found to be valuable in the evaluation of efficacy after radiotherapy. Mongula et al. found that the use of [^18^F]-FDG PET/MRI in patients with cervical cancer after radiotherapy could affect the treatment strategy in 50% of patients [103]. Kovács et al. established a mouse model to demonstrate that SUV and ADC values in [^18^F]-FDG PET/MRI can be used to determine physiological changes in brain tissue after radiotherapy and thus monitor the occurrence of adverse effects after radiotherapy, making [^18^F]-FDG PET/MRI an irreplaceable tool in the assessment of efficacy after radiotherapy [104]. However, unlike post-chemotherapy efficacy evaluation, radiotherapy may yield up to 50% false positives owing to the occurrence of radiation-induced fibrosis, necrosis, inflammation, and edema [105, 106], potentially limiting the role of [^18^F]-FDG PET/MRI in assessing radiotherapy efficacy. Nevertheless, recent studies suggest that functional imaging techniques, such as diffusion kurtosis imaging (DKI) and ^11^C-methionine (^11^C-MET)-PET, may be beneficial in differentiating residual tumors from radiation-induced fibrotic tissue [103, 107].

In addition, ultrafast DCE-MRI, advanced DWI sequences such as intravoxel incoherent motion (IVIM), diffusion tensor imaging (DTI), proton MR spectroscopy (^1^H MRS), and chemical exchange saturation transfer (CEST) imaging have shown promise in evaluating the efficacy of cancer patients [108–110]. For instance, due to special IVIM parameters, PET/IVIM-MRI, which simultaneously displays the diffusion of water molecules and microcirculation perfusion in the tumor tissue, can be used to evaluate early treatment outcomes for locally advanced cervical cancer and to predict lymphovascular space invasion in cervical cancer without lymphatic metastasis [86, 93, 111]. However, it is noteworthy that the use of these novel imaging sequences in radiotherapy requires further investigation.

## Patient surveillance

Patient surveillance plays a key role in the survival of cancer patients. Local recurrence may be the main cause of treatment failure, so timely and accurate restaging of patients with suspected recurrence is required for optimal management. Traditional imaging methods play a decisive role in the evaluation of locoregional recurrence. However, the anatomical changes, scars, and radiation-induced inflammation caused by radiotherapy or surgery make it difficult to distinguish the active tumor tissue, leading to incorrect restaging and thus affecting further treatment decisions [112–114]. [^18^F]-FDG PET/MRI is expected to be the best choice for long-term follow-up of patients. Sawicki et al. showed that in patients with suspected pelvic tumor recurrence, [^18^F]-FDG PET/MRI correctly diagnosed 100% of malignant lesions, compared with MRI only 74.6%. PET/MRI was also found to reduce the probability of misdiagnosing distant metastases as only local recurrence, thus leading to a higher tumor stage [115]. Since PET/CT was introduced, it has had a high utilization rate in patient follow-up due to its combination of metabolic and anatomical information [116–118]. Some studies have compared [^18^F]-FDG PET/MRI with PET/CT and found that there was no difference in the diagnostic performance of the two in follow-up, but [^18^F]-FDG PET/MRI can better define the tumor margin, which could show more unclear FDG findings [119]. Additionally, during long-term follow-up, patients may undergo repeated radiation exposure from CT and PET/CT scans, which has been shown to potentially elevate the risk of cancer [120, 121]. Notably, PET/MRI offers a radiation-free alternative, mitigating the potential for cumulative radiation damage and alleviating patient concerns. However, the high purchase and maintenance costs and potential reimbursement issues in PET/MRI could limit its use.

## Other works

### Specific PET tracers

With the introduction of various tracers, PET/MRI can provide complementary and sensitive information for lesion characterization, boosting its utility in RT planning. As a hypoxic PET tracer, [^18^F]-Fluoromisonidazole([^18^F]-FMISO) can predict radiotherapy response, given that normoxic tumors respond better to treatment than hypoxic ones [122]. Neuroendocrine tumors (NETs) frequently overexpress somatostatin receptors (SSTRs). Thus in RT planning of NETs, SSTR-targeted molecular imaging, such as [^68^Ga]-DOTATOC, [^68^Ga]-DOTATATE, or [^68^Ga]-DOTANOC, was used to precisely locate and delineate tumor targets [59]. Recently, the texture features of [^68^Ga]-DOTATOC-PET/MRI have also been increasingly used to evaluate treatment-related changes in NETs [123]. In addition, as PSMA is highly expressed in prostate cancer (PCa) cells, ^68^Ga-PSMA-11 PET/MRI provides a good detection rate for PCa in biochemical recurrence after initial curative therapy [124]. Other promising tracers, such as fibroblast activation protein inhibitor-PET/MRI(FAPI-PET/MRI) [125] and ^18^F-EF5-PET/MRI [126], are currently under investigation but not yet utilized in routine clinical radiotherapy.

### Multimodality nanoparticle probes

When the lesion is not well defined from the surrounding tissue, MRI contrast agents are often used to improve image contrast. In integrated PET/MRI, PET tracer and MRI contrast agent are initially used in combination; however, this combination increases both the number of injections and the time spent by patients in the scanner and may ultimately increase costs [127, 128]. To solve these problems, dual-modality probes are under development. Nanoparticles (NPs) lay the foundation for the realization of dual-modality probes due to their highly tunable characteristics and large surface area: volume [129]. In the past few years, iron oxide nanoparticles have been radiolabeled with different radioisotopes (^18^F, ^11^C, ^13^N, ^15^O, ^124^I, ^64^Cu, ^68^Ga) for cancer diagnosis and evaluation in PET/MRI [130–132]. Recently, ^68^Ga-magnetic iron oxide nanoparticles targeting PSMA and gastrin-releasing peptide receptors (GRPRs) have been developed as a potential tool for PET/MRI diagnosis of PCa and are thought to improve the efficacy of PCa-targeted therapy, but further application in mouse models is needed [128]. In addition, multimodality nanoparticle probes can be concurrently delivered with drugs or therapeutic agents gathering a dual diagnostic and therapeutic effect to perform cancer diagnosis and treatment at the same time [129].

## Limitations

Compared with other imaging modalities, although PET/MRI has its outstanding advantages, it still has some inevitable drawbacks. For instance, the inherent drawbacks of PET/MRI make it limiting for some patients. Firstly, a longer image acquisition time makes it less suitable for elderly and pediatric patients who may struggle to remain still for extended periods. Secondly, due to the narrower and longer bore diameter of PET/MRI, some patients who are larger or obese are not candidates for PET/MRI. Thirdly, patients with metal implants, ferromagnetic objects, etc. cannot use PET/MRI, as their presence distorts the magnetic fields and adversely affects image quality. In addition, while the simultaneous collection of PET and MRI data offers potential advantages in motion correction compared to PET/CT, motion artifacts remain an issue. Static motion correction techniques have been suggested as a possible improvement, but these techniques still require further validation [133]. Another notable limitation is the inferiority of MRI compared to CT in the assessment of pulmonary nodules due to the low proton density, magnetic susceptibility, and respiratory motion of lung tissue. Moreover, PET faces difficulties in detecting nodules with low glucose metabolism. Therefore, the precise application of PET/MRI in lung cancer remains an area to be explored [15, 134, 135]. The comparison between PET/MRI and other imaging methods at different angles was shown in Table 3.

**Table 3:**
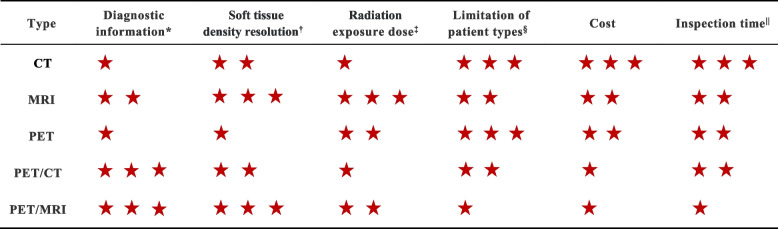
Comparison of different imaging methods

Red five-pointed stars represent the effect of the image: Three stars indicate a good effect and could be considered the first choice for patients. Two stars indicate a moderate effect and could be considered the second choice for patients. One star indicates a poor effect and could be considered the third choice for patients

^*^Diagnostic information: CT could obtain tumor anatomy and morphology information; MRI could obtain anatomy, morphology, and physiology information; PET could obtain tumor physiology and molecular information; PET/CT could obtain tumor anatomy, morphology, physiology, and molecular information; PET/MRI could obtain tumor anatomy, morphology, physiology, and molecular information

^†^Soft tissue density resolution: MRI is better than CT for soft tissue with more water. On PET, which reflects molecular metabolism, lesions usually show high metabolism and poor recognition of morphological structure

^‡^Radiation exposure dose: The radiation dose of CT is mainly from ionizing radiation, and the radiation dose of PET is mainly from radioactive tracers, while the radiation exposure dose of CT examination is higher than that of radio pharyngology. MRI produces no radiation. The radiation dose of PET/MRI mainly comes from PET. Most of the radiation dose in PET/CT comes from the CT, and a small part comes from the PET radiotracer

^§^Limitation of patient types: CT examination has radiation, so pregnant women and children should try to avoid using it. However, since it only takes a few minutes, most patients can tolerate it, and it can be used as the first choice in emergencies. MRI, PET, PET/CT, and PET/MRI take a long time to examine, which is unsuitable for emergency patients or some people who find it difficult to stay still for a long time. In addition, PET requires injection of radioactive drugs to image, so it is unsuitable for patients with drug allergies. MRI is noisy and narrow, which is not friendly for claustrophobic or obese patients and unsuitable for patients carrying metal objects. PET/CT and PET/MRI are integrated imaging modalities and, therefore, have the same problems as individual imaging modalities. ^||^Inspection time: CT usually takes a few minutes. MRI, PET, and PET/CT take about 30 min. PET/MRI takes an hour or longer

## Outlook

Integrated PET/MRI can get both the shape of the tumor and its biological characteristics simultaneously, allowing for a precise understanding of the disease, which in turn guides the implementation of the radiotherapy plan more precisely. With the advent of new technologies, it is anticipated that PET/MRI will be utilized more frequently for radiotherapy.

## Data Availability

Not applicable.

## Abbreviations

[^18^F]-FDG: ^18^F-fluorodeoxyglucose
[^18^F]-FMISO: [^18^F]-Fluoromisonidazole
^11^C-MET: ^11^C-methionine
^1^H MRS: Proton MR spectroscopy
3D-CRT: Three-dimensional conformal radiotherapy
AC: Attenuation correction
ADC: Apparent diffusion coefficient
AI: Artificial intelligence
aTL: Adaptive threshold level
BTV: Biological target volume
CAD: Computer-aided design
CEST: Chemical exchange saturation transfer
CMR: Complete metabolic remission
CNNs: Convolutional neural networks
CTV: Clinical tumor volume
DCE: Dynamic contrast-enhanced
DKI: Diffusion kurtosis imaging
D_min_: Minimum diffusion correlation coefficient
DSC: Dice similarity coefficient
DTI: Diffusion tensor imaging
DWI: Diffusion-weighted imaging
FAPI-PET/MRI: Fibroblast activation protein inhibitor-PET/MRI
FLAIR: Fluid-attenuated inversion recovery
FOV: Field of view
GRPRs: Gastrin-releasing peptide receptors
GTV: Gross tumor volume
GTVnd: Nodal gross tumor volume
IMRT: Intensity-modulated radiation therapy
IR-GRE: Inversion recovery gradient echo
IVIM: Intravoxel incoherent motion
*K*_ep_: Rate constant
*K*^trans^: Volume transfer constant
MRI-UTE: Magnetic resonance imaging-ultra short echo time
MTV: Metabolic tumor volume
NETs: Neuroendocrine tumors
NPs: Nanoparticles
OAR: Organs at risk
PCa: Prostate cancer
PET/MRI: Positron emission tomography/magnetic resonance imaging
PR: Partial response
PSMA: Prostate-specific membrane antigen
PTV: Planning target volume
RF: Radiation frequency
RT: Radiotherapy
s-CT: Substitute CT
SD: Stable disease
SI: Signal strength
SNR: Signal-to-noise ratio
SRT: Stereotactic radiotherapy
SSTRs: Somatostatin receptors
SUV: Standardized uptake value
SUV_max_: Maximum standardized uptake value
T1WI: T1-weighted imaging
T2WI: T2-weighted imaging
TE: The time of echo
TLG: Total lesion glycolysis
TSE: Turbo spin-echo
ΔD_mean_: The percentage changes of the mean diffusion-related coefficient
ΔD_min_: The percentage changes in minimum diffusion-related coefficient
ΔSUV_max_: The percentage changes of the maximum standardized uptake value
